# Human-Machine Agreement in Medical Ethics: Patient Autonomy Case-Based Evaluation of Large Language Models

**DOI:** 10.2196/77061

**Published:** 2025-10-23

**Authors:** Vamshi Mugu, Brendan Carr, Ashish Khandelwal, Mike Olson, John Schupbach, John Zietlow, T N Diem Vu, Alex Chan, Christopher Collura, John Schmitz

**Affiliations:** 1 Department of Radiology Mayo Clinic Rochester, MN United States; 2 Department of Emergency Medicine Mayo Clinic Rochester, MN United States; 3 Department of Trauma, Critical Care, and General Surgery Mayo Clinic Rochester, MN United States; 4 Department of Neonatal Medicine Mayo Clinic Rochester, MN United States

**Keywords:** large language models, medical ethics, patient autonomy, generative AI, human in the loop

## Abstract

**Background:**

Medical ethics provides a moral framework for the practice of clinical medicine. Four principles, that is, beneficence, nonmaleficence, patient autonomy, and justice, form the cornerstones of medical ethics as it is practiced today. Of these 4 principles, patient autonomy holds a pivotal position and often takes precedence in ethical dilemmas that result from conflicts among the 4 principles. Its importance serves as a constant reminder to the clinician that the “needs of the patient come first.” With their remarkable ability to process natural language, large language models (LLMs) have recently pervaded nearly every aspect of human life, including medicine and medical ethics. Reliance on tools such as LLMs, however, poses fundamental questions in medical ethics, where human-like reasoning, emotional intelligence, and an understanding of local context and values are of utmost importance.

**Objective:**

While emphasizing the central role of the human factor, we undertake a bold venture to establish some confidence in LLMs, as it pertains to medical ethics by not only evaluating the status quo of foundational LLMs but also exploring ways to improve the LLMs by using patient autonomy–based hypothetical cases. Although literature today is certainly lacking in such ventures, we also believe projects such as ours must be frequently revisited in the field of LLMs, which is evolving at a pace that is both rapid and unprecedented.

**Methods:**

We evaluated 3 foundational LLMs (ChatGPT, LLaMA, and Gemini) on hypothetical cases in patient autonomy. We used Cohen κ to compare LLM responses to the consensus from a physician panel. McNemar test was used during the improvement phase and to report the final significance of improved agreement of each LLM with physician consensus. *P* values less than .05 were considered significant. An agreement with κ<0 was designated as poor, 0-0.2 as slight, 0.2-0.4 as fair, 0.41-0.6 as moderate, 0.61-0.8 as substantial, and 0.81-1 as almost perfect.

**Results:**

There was slight to fair agreement between the foundational LLMs and the physician consensus. With iterative improvement techniques, this agreement evolved to be substantial or higher (Cohen κ of 0.73-0.82). The degree of improvement was statistically significant (*P*=.006 for ChatGPT, *P*<.001 for Gemini, and *P*<.001 for LLaMA).

**Conclusions:**

Although LLMs hold great potential for use in medicine, there needs to be an abundance of caution in using foundational LLMs in domains such as medical ethics. With adequate human oversight in testing and utilizing established techniques, LLM responses can be better aligned to human responses, even in the domain of medical ethics.

## Introduction

The American Medical Association defines medical ethics as a moral framework for the practice of clinical medicine [1]. Proposed by Beauchamp and Childress [2], the 4 principles, that is, beneficence, nonmaleficence, patient autonomy, and justice, are often regarded as the cornerstones of medical ethics as the field is taught and practiced today. Among these 4 principles, patient autonomy holds a particularly important place due to its frequent precedence over the other 3 principles when an ethical dilemma arises due to conflict among the 4 principles [3].

Large language models (LLMs) represent a new paradigm in artificial intelligence with remarkable abilities to process and generate text in natural language [4]. Foundational LLMs can perform many different tasks but sometimes lack domain-specific capabilities [5]. Although the abilities of foundational LLMs have translated into numerous applications in medical education, research, and practice [6,7], concerns remain about their accuracy, biases, and potential misuse [8]. A recurrent theme in proposed approaches to address these concerns is the importance of human expert participation in the evaluation and governance of LLMs. This importance of the human factor, which is also emphasized in this study, has prompted the development of guidelines from various governing bodies across continents [9-11].

There is a notable scarcity of literature on the role of LLMs in medical ethics, particularly on ways to improve the reliability of foundational LLMs on tasks specific to medical ethics. The comparative evaluation of LLMs in medical ethics is nearly nonexistent, with only ChatGPT being evaluated to any considerable extent [12]. Although the body of knowledge, particularly prompt engineering [13], to elicit improved responses from LLMs in general is enlarging, the discussion of these techniques in the context of medical ethics is lacking. We venture on a bold undertaking to not only evaluate the foundational LLMs in their ability to analyze cases in patient autonomy but also explore techniques to improve the reliability of LLM responses, thereby attempting to increase trust in the technology while emphasizing the indispensable role of the human factor.

## Methods

### Ethical Considerations

After obtaining approval from the Mayo Clinic Institutional Review Board (which also governs research ethics per approval 25-001042), 44 hypothetical cases in patient autonomy requiring yes or no responses were composed. No real patient information was used for this research. The first author adopted these cases from the literature [14] and from personal and collective clinical experiences, with a focus on capacity to consent, occupational exposure, confidentiality, informed consent for a minor patient, patient preferences, treatment refusal, and training needs.

### Hypothetical Case

A hypothetical case is presented in Textbox 1. The LLMs and the physicians on the panel (described subsequently) were blinded to the sources of the cases and others’ responses.

**Sample hypothetical case. Note that the hypothetical case deals with the capacity to consent and treatment refusal.**

**Sample hypothetical case**

David is a 79-year-old male with a history of myocardial infarction whose wife, Mary called 911 about David’s chest pain. The pain started 30 minutes ago and is similar to some of his prior episodes which needed admission to a hospital. Upon arrival of the emergency medical services (EMS) team, David refuses any intervention. David seems distressed from pain but is orientedx3.

Should the EMS team take David to the nearest hospital?

Three foundational LLMs were chosen for this study: ChatGPT version 4o (ChatGPT), LLaMA 3.1 70b BF16 (LLaMA), and Gemini 1.5 (Gemini). Although the open-source LLaMA was used locally with Ollama version 0.1.33 (Ollama, Inc), the 2 closed-source models, ChatGPT and Gemini, were accessed using their respective web interfaces. Where applicable, the default parameters (eg, temperature of 0.8, top_p of 0.95 for Ollama) were used. There were no concerns for Health Insurance Portability and Accountability Act (HIPAA) noncompliance due to the hypothetical nature of the cases. Five physicians (P1-P5) with respective board certifications from emergency medicine, surgery, and radiology comprised the physician panel.

### Study Design

**Figure 1:**
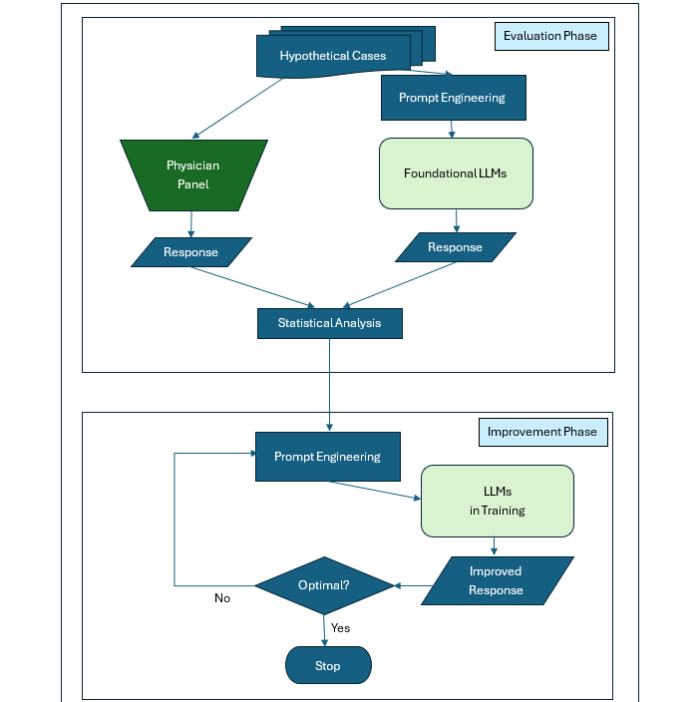
Two-phase design with evaluation phase and improvement phase. The evaluation phase compares the responses of foundational LLMs to physician panel consensus. The improvement phase is designed to improve the agreement between the LLMs and the physician panel consensus. LLM: large language model.

Our study was designed in 2 phases (Figure 1): evaluation phase and improvement phase. The evaluation phase was designed to compare the responses from foundational LLMs to those from the physician panel, while the improvement phase was designed to optimize the responses from LLMs to improve agreement with the physician panel. The LLMs and the physicians on the panel were blinded to each other’s responses. Interobserver agreement among the physicians on the panel and among the foundational LLMs was calculated using Fleiss κ. Cohen κ was used to compare the responses of each LLM with the physician consensus, which was defined as the majority response from the physicians in the panel. A missing value from an LLM was considered “No” for statistical analysis purposes. For illustration, pairwise agreements were depicted as a heatmap and listed as proportions in a tabular format. Python statsmodels (version 0.15.0) was used for statistical testing. An agreement with κ<0 was considered to be poor, 0 to 0.2 was considered slight, 0.21 to 0.4 as fair, 0.41 to 0.6 as moderate, 0.61 to 0.8 as substantial, and 0.81 to 1 as almost perfect. Although we reserved several prompt engineering techniques to employ during the improvement phase, we nevertheless used some techniques such as role playing, forcefulness, and chain-of-thought during the evaluation phase [13].

During the improvement phase, prompt engineering techniques such as chain-of-thought, N-shot prompting, directional stimulus, versioning, rephrase-and-respond, and long context prompting were used to improve LLM responses [13,15-18]. The goal of this iterative process was to improve LLMs to produce responses that were better aligned to the physician consensus, reducing any statistically significant differences. The stopping point of the iterative process was determined to be when no more reduction in the number of differing responses could be achieved. The degree of improvement was assessed using McNemar test. A *P* value less than .05 was considered significant.

### Sample Human-Model Interaction

A sample human-model interaction during the evaluation phase and the improvement phase is provided in Textbox 2. This hypothetical case deals with consenting for a minor patient and demonstrates human-model interaction during the evaluation and improvement phases. The use of 2 prompt engineering techniques, that is, question-refinement and chain-of-thought, is illustrated with this sample.

**Sample hypothetical case with human-model interaction during the evaluation and improvement phases. Two prompt engineering techniques, that is, question-refinement and chain-of-thought, are demonstrated here.**

**Sample interaction with a model demonstrating evaluation and improvement phases**

**Hypothetical case:** Rachel is a 9-year-old girl from Virginia. She fell at the school basketball court and was taken to the emergency department by the teacher. She is found to have an elbow fracture that needs nonemergent surgical fixation. Can the surgeon proceed with the surgery?

**Interaction snippet during the evaluation phase**

Gemini’s response

Answer: Yes

Thought Process:

1. The surgeon has a duty to act in the best interest of the patient. In this case, the surgery is necessary to treat the elbow fracture and prevent complications.
2. Delaying the surgery could lead to worsening of the condition, pain, and potential long-term disability.
3. Therefore, proceeding with the surgery is in the patient's best interest.

**Interaction snippet during the improvement phase**

Refined question (provided by ChatGPT upon human request): Given that Rachel, a 9-year-old girl from Virginia, fell at the school basketball court and was taken to the emergency department by her teacher, where she was diagnosed with an elbow fracture that requires nonemergent surgical fixation, does the surgeon have the necessary consent and authority to proceed with the surgery?

Gemini’s response

Answer: No

Thought Process:

1. Rachel is a minor and therefore lacks the capacity to provide informed consent for the surgery.
2. Her legal guardian, likely her parents, must provide consent.

## Results

**Figure 2:**
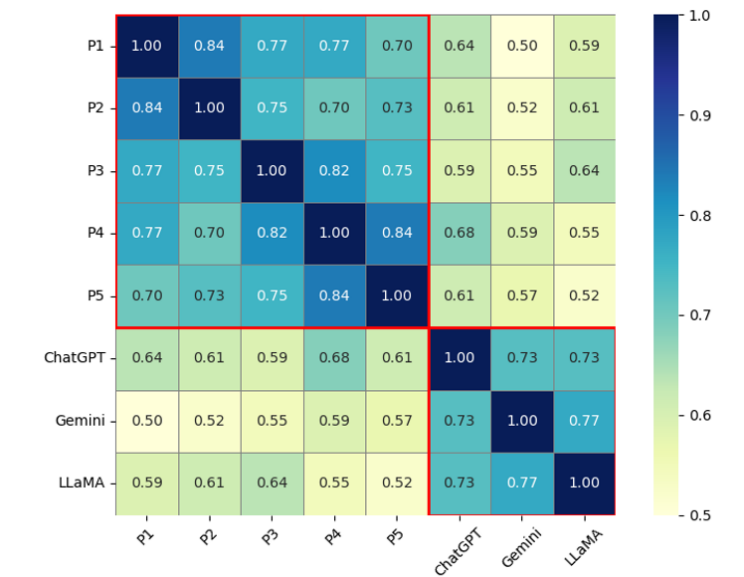
Pairwise agreement heatmap for illustration purposes only. Note the substantial agreement among large language models and among physicians on the panel (P1-P5) during the evaluation phase (enclosed by red boxes). The improvement phase (discussed later) is however designed to improve the agreement of large language models with physician consensus, not with individual physician responses.

Of the 44 cases, 6 focused on informed consent, 6 on capacity to consent, 6 on occupational exposure, 6 on training needs, 7 on treatment refusal, 6 on patient preferences, and 7 on confidentiality. A sample hypothetical case is presented in Textbox 1. There was substantial agreement among the physicians in the panel (κ=0.77). There was also substantial agreement among the foundational LLMs (κ=0.73). There was slight agreement between the foundational Gemini and the physician consensus (κ=0), slight agreement between the foundational LLaMA and the physician consensus (κ=0.14), and fair agreement between the foundational ChatGPT and the physician consensus (κ=0.32). Pairwise agreement is depicted in Figure 2 as a heatmap for illustration purposes. However, note that the Improvement phase was designed to improve the agreement between LLMs and the physician panel consensus rather than individual physician responses.

At the end of the improvement phase, agreement between all the improved LLMs and the physician consensus was substantial or higher (κ=0.77, 95% CI 0.59-0.96 for ChatGPT; κ=0.73, 95% CI 0.52-0.93 for Gemini; and κ=0.82, 95% CI 0.65-0.99 for LLaMA). The degree of improvement for all the LLMs was statistically significant (*P*=.006 for ChatGPT, *P*<.001 for Gemini, and *P*<.001 for LLaMA). The degree of improvement is listed in Table 1.

**Table 1:** Proportion of agreement between each large language model and physician consensus before and after improvement. Note that all large language models underwent improvement that was statistically significant.

Model	Before improvement, n	After improvement, n	Degree of improvement (N=44), n (%)	*P* value
ChatGPT	27	39	+12 (27)	.006
Gemini	22	38	+16 (36)	<.001
LLaMA	26	40	+14 (32)	<.001

## Discussion

### Principal Findings

Medical ethics provides a moral framework for the practice of clinical medicine, particularly as it pertains to physician responsibilities and obligations in the context of patient-physician interactions [1]. Beauchamp and Childress [2] are often credited for laying the foundation for this framework with 4 principles: beneficence, nonmaleficence, patient autonomy, and justice. Although these principles have been extensively criticized [19], debated [20], and defended [21], they nevertheless remain at the center of modern teaching and practice of medical ethics. The principle of patient autonomy holds a particularly important place among the four, often taking precedence over the other 3 principles when an ethical dilemma arises due to conflicts among the 4 principles [3]. It is a constant reminder to the medical practitioner that “the patient must remain at the heart of his or her own individual battle” [22] and that “the needs of the patient come first” [23].

Despite their relative infancy, LLMs have revolutionized various aspects of health care, ranging from medical education to practice and research [6,7]. LLMs have demonstrated remarkable accuracy on tasks that were once believed to be germane to the rigorously trained human, such as outperforming medical students, and neurosurgery residents on neurosurgery written board-like questions [24]. LLMs are nevertheless prone to limitations, a few of which are particularly concerning in medicine, such as the fabrication of responses not based on evidence—a behavior referred to as hallucination [25]. Moreover, reliance on technology such as LLMs poses fundamental questions in medical ethics, where human-like reasoning, emotional intelligence, and an understanding of local context and values are of utmost importance [26,27]. A recurrent theme in the proposed approaches to address these concerns is the importance of human expert participation in evaluation and governance of LLMs. This importance of the human factor, which is also emphasized in our study, has prompted the development of guidelines from various governing bodies across continents [9-11] and paved the way for effective interaction such as through prompt engineering [13]. The field of prompt engineering is rapidly evolving with several successful approaches already being adopted, including chain-of-thought, directional stimulus, forcefulness, self-correction and consistency, role playing, reflection, guiding output with rails, and long context prompting [13,15-18].

Although literature on the employment of artificial intelligence in the domain of medical ethics is sparse, some work warrants mention. Before the conception of LLMs, Shalowitz et al [28] proposed a population-based treatment indicator and claimed its superiority to traditional surrogate decision-making in treatment decisions made for incapacitated patients [28]. The population-based treatment indicator was critiqued for disrespecting patient autonomy by relying on statistical evidence rather than individual patient preferences [26]. Leveraging the natural language processing power of LLMs, Earp et al [29] proposed P4, a personalized patient preference predictor, to address this specific concern [29]. P4’s claim of superiority is based on its incorporation of material from prior patient treatment decisions, thereby creating a “digital twin” to act on behalf of the incapacitated patient, when such a need arises.

Our study has perhaps the closest resemblance to the expert panel evaluation of GPT-4 by Balas et al [27] who created a set of 8 ethical case vignettes and present to the LLM via a priori prompt template. LLM responses are then evaluated for the depth of reasoning, ability to consider alternate viewpoints, and sensitivity to nuances of ethical dilemmas. Although our case repertoire is considerably larger, it is also arguably simpler since we do not probe the depth of reasoning. However, the greatest distinction from their study is in the improvement phase. In the strictest sense of the study design, their study can be considered as lacking this phase. By not only evaluating the foundational LLMs but also exploring the ways to improve their performance as it pertains to a subset of medical ethics, we venture to establish some confidence in this promising technology while reminding the adopters of the importance of the human factor.

### Limitations

This study was subject to a few limitations. Although larger than other similar studies, it still involved a limited set of questions covering a relatively small section of medical ethics. It is possible that LLMs could perform differently with different clinical scenarios or ethical topics. Although we employed several types of prompt engineering, other improvement techniques such as RAG and fine-tuning were not used. Over time, work will undoubtedly continue to improve the performance of foundational LLMs in medical ethics; how quickly and whether the need for human supervision will ever be entirely eliminated remains to be seen.

### Conclusion

The use of foundational LLMs in domains such as medical ethics warrants an abundance of caution and intricate involvement of a human expert. With adequate testing and by utilizing established techniques such as prompt engineering, LLM performance can be improved, even in the domain of medical ethics, where human-like reasoning, emotional intelligence, and context awareness are crucial.

## Abbreviations

HIPAA: Health Insurance Portability and Accountability Act
LLM: large language model

## References

[1] Medical ethics. American Medical Association.

[2] Beauchamp TL, Childress JF (2019). Principles of Biomedical Ethics (Eighth Edition).

[3] Badger J, Ladd RE, Adler P (2009). Respecting patient autonomy versus protecting the patient's health: a dilemma for healthcare providers. JONA's Healthcare Law, Ethics, and Regulation.

[4] Naveed H, Khan AU, Qiu S, Saqib M, Anwar S, Usman M, Akhtar N, Barnes N, Mian A (2025). A comprehensive overview of large language models. ACM Trans Intell Syst Technol.

[5] Scott IA, Zuccon G (2024). The new paradigm in machine learning - foundation models, large language models and beyond: a primer for physicians. Intern Med J.

[6] Cascella M, Montomoli J, Bellini V, Bignami E (2023). Evaluating the feasibility of ChatGPT in healthcare: an analysis of multiple clinical and research scenarios. J Med Syst.

[7] Sallam M (2023). ChatGPT utility in healthcare education, research, and practice: systematic review on the promising perspectives and valid concerns. Healthcare (Basel).

[8] Kwong J, Wang Serena C Y, Nickel Grace C, Cacciamani Giovanni E, Kvedar Joseph C (2024). The long but necessary road to responsible use of large language models in healthcare research. NPJ Digit Med.

[9] Biden J Executive order on the safe, secure, and trustworthy development and use of artificial intelligence. The White House.

[10] Déclaration de Montréal IA Responsable.

[11] Ethics guidelines for trustworthy AI. European Commission.

[12] Skryd A, Lawrence K (2024). ChatGPT as a tool for medical education and clinical decision-making on the wards: case study. JMIR Form Res.

[13] Amatriain X (2024). Prompt design and engineering: introduction and advanced methods. ArXiv. Preprint posted online on May 5, 2024.

[14] Johnston C, Bradbury P (2016). 100 Cases in Clinical Ethics and Law (Second Edition).

[15] Li Z, Peng B, He P, et al (2023). Guiding large language models via directional stimulus prompting. ArXiv. Preprint posted on October 9, 2023.

[16] Chang K, Xu S, Wang C, et al (2024). Efficient prompting methods for large language models: a survey. ArXiv. Preprint posted on December 2, 2024.

[17] Parthasarathy V, Zafar A, Khan A, Shahid A (2024). The ultimate guide to fine-tuning llms from basics to breakthroughs: an exhaustive review of technologies, research, best practices, applied research challenges and opportunities. ArXiv. Preprint posted on August 23, 2024.

[18] Brown TB, Mann B, Ryder N, et al (2020). Language models are few-shot learners. ArXiv. Preprint posted on May 28, 2020.

[19] Clouser K, Gert B (1990). A critique of principlism. J Med Philos.

[20] Davis R (1995). The principlism debate: a critical overview. J Med Philos.

[21] Gillon R (1995). Defending 'the four principles' approach to biomedical ethics. J Med Ethics.

[22] Mattei JF (2018). [Respecting the patient's autonomy]. Soins.

[23] Beck CS (2000). The needs of the patient come first. Mayo Clinic Proceedings.

[24] Guerra G, Hofmann Hayden, Sobhani Sina, Hofmann Grady, Gomez David, Soroudi Daniel, Hopkins Benjamin S, Dallas Jonathan, Pangal Dhiraj J, Cheok Stephanie, Nguyen Vincent N, Mack William J, Zada Gabriel (2023). GPT-4 artificial intelligence model outperforms ChatGPT, medical students, and neurosurgery residents on neurosurgery written board-like questions. World Neurosurg.

[25] Májovský Martin, Černý Martin, Kasal M, Komarc M, Netuka D (2023). Artificial intelligence can generate fraudulent but authentic-looking scientific medical articles: Pandora's box has been opened. J Med Internet Res.

[26] Sharadin N (2018). Patient preference predictors and the problem of naked statistical evidence. J Med Ethics.

[27] Balas M, Wadden JJ, Hébert Philip C, Mathison E, Warren MD, Seavilleklein V, Wyzynski D, Callahan A, Crawford SA, Arjmand P, Ing EB (2024). Exploring the potential utility of AI large language models for medical ethics: an expert panel evaluation of GPT-4. J Med Ethics.

[28] Shalowitz D, Garrett-Mayer Elizabeth, Wendler David (2007). How should treatment decisions be made for incapacitated patients, and why?. PLoS Med.

[29] Earp BD, Porsdam Mann S, Allen J, Salloch S, Suren V, Jongsma K, Braun M, Wilkinson D, Sinnott-Armstrong W, Rid A, Wendler D, Savulescu J (2024). A personalized patient preference predictor for substituted judgments in healthcare: technically feasible and ethically desirable. Am J Bioeth.

